# Was federal parity associated with changes in Out-of-network mental health care use and spending?

**DOI:** 10.1186/s12913-017-2261-9

**Published:** 2017-05-02

**Authors:** Susan H. Busch, Emma E. Mcginty, Elizabeth A. Stuart, Haiden A. Huskamp, Teresa B. Gibson, Howard H. Goldman, Colleen L. Barry

**Affiliations:** 1 Department of Health Policy and Management, Yale School of Public Health, 60 College Street, New Haven, CT 06520-8034 USA; 2 Department of Health Policy and Management, Johns Hopkins Bloomberg School of Public Health, Baltimore, MD USA; 3 Department of Mental Health, Johns Hopkins Bloomberg School of Public Health, Baltimore, MD USA; 4 Department of Biostatistics, Johns Hopkins Bloomberg School of Public Health, Baltimore, MD USA; 5 Department of Health Care Policy, Harvard Medical School, Boston, MA USA; 6 Truven Health Analytics, Ann Arbor, MI USA; 7 Department of Psychiatry, University of Maryland, Baltimore, MD USA

## Abstract

**Background:**

The goal of the Paul Wellstone and Pete Domenici Mental Health Parity and Addiction Equity Act is to eliminate differences in insurance coverage between behavioral health and general medical care. The law requires out-of-network mental health benefits be equivalent to out-of-network medical/surgical benefits. Insurers were concerned this provision would lead to unsustainable increases in out-of-network related expenditures. We examined whether federal parity implementation was associated with significant increases in out-of-network mental health care use and spending.

**Methods:**

We conducted an interrupted time series analysis using health insurance claims from self-insured employers (2007–2012). We examined changes in the probability of using out-of-network mental health services and, conditional on out-of-network mental health service use, changes in the number of outpatient out-of-network mental health visits and total out-of-network mental health spending associated with the implementation of federal parity in 2010.

**Results:**

From 2007 to 2012, the proportion of individuals receiving any out-of-network mental health services each month declined dramatically from 18 to 12%, with a one-time drop of 3 percentage points at parity implementation (*p* < .01). Among out-of-network mental health service users, there was an increase in the number of visits per month (.12 visits; *p* < .01) and total spending per month ($49; *p* < .01) at parity implementation. Although there was a one-time increase in spending at parity implementation, this increase was accompanied by an attenuation of a trend toward increased spending growth, such that spending was back to original predictions by the end of our study period.

**Conclusions:**

Despite concerns expressed by the health insurance industry when federal parity was enacted, out-of-network mental health spending did not substantially increase after parity implementation. In addition, use of out-of-network mental health services appears to have contracted rather than expanded, suggesting insurers may have implemented other policies to curb out-of-network use, such as increasing access to in-network providers.

**Electronic supplementary material:**

The online version of this article (doi:10.1186/s12913-017-2261-9) contains supplementary material, which is available to authorized users.

## Background

The Paul Wellstone and Pete Domenici Mental Health Parity and Addiction Equity Act of 2008 (MHPAEA) requires those in the large group market (i.e., firms with 50 or more employees) covering mental health services to offer benefits for those services on par with benefits for medical/surgical services. Prior to implementation of this law in 2010, health insurers often covered mental health services with higher cost sharing and deductibles compared with medical/surgical services and with special annual treatment limits (i.e., annual inpatient day limits or outpatient visit limits). In addition, the federal parity law required plans that offer an out-of-network benefit for medical/surgical care to have equivalent out-of-network benefits for behavioral health care. As clarified in interim final regulations released in February 2010 [1], plans are also prohibited from using different standards for choosing or reimbursing in-network mental health providers than those used for choosing or reimbursing in-network medical/surgical providers. Provisions related to out-of-network care were included in federal parity due to concerns that plans would restrict access to mental health providers by establishing networks that did not include providers with appropriate clinical training or accessible and valid contact information, making it difficult for patients to access services in-network [2, 3] or that plans would use limits on the out-of-network benefit to discourage potentially high cost patients from choosing their plan. Financial burden was also a concern. Out-of-network providers often balance bill patients, charging patients the difference between provider list price and insurer reimbursement [4]. When combined with exceptionally high cost sharing, the financial consequences for patients may be extreme [5].

These concerns developed due to evidence of limited access to in-network providers after implementation of past parity laws. In 2001, the Federal Employee Health Benefit (FEHB) program implemented parity, providing insurance coverage for in-network mental health benefits equivalent to in-network general medical benefits. Out-of-network benefit requirements did not change, and mental health out-of-network benefits often continued to have greater cost sharing than general medical out-of-network benefits. One study of Washington DC area mental health providers 4 years after implementation of the FEHB program parity provision found that the majority of providers studied did not participate in any managed care network, and among providers who did participate, only 73–85% were accepting new patients, suggesting the importance of access to out-of-network providers [6]. Yet, requiring equivalent out-of-network benefits was untested since most prior state and smaller-scope parity laws did not extend to out-of-network benefits. Health plans were concerned this provision would reduce their ability to use managed care techniques to control utilization in response to parity [7].

The use of out-of-network care is common in mental health. One national survey conducted in February 2011 (1-year after the initial implementation of federal parity), found that, among individuals who used health care services in a given year, the proportion using out-of-network care was almost three times higher for mental health care services compared with general health care services (18 versus 7%) [8]. An analysis of claims from one insurer found that 16% of individuals using mental health services used at least some out-of-network services, and that individuals who used out-of-network care typically used more services and were more likely to have serious mental illness [9]. The high proportion of mental health care services provided by out-of-network providers led to continuing concerns about adequate access to in-network providers.

The objectives of this paper are to examine whether federal parity was associated with: 1) Changes in the proportion of patients who used mental health services who saw at least one out-of-network mental health provider; 2) the mean number of out-of-network outpatient visits, among patients who saw an out-of-network provider; 3) total out-of-network mental health spending, among patients who saw an out-of-network provider.

## Methods

### Data

We used the *Truven Health MarketScan Database* (2007–2012) to examine changes in use and spending on out-of-network mental health care services before (2007–2009) and after (2010–2012) federal parity implementation. These data include health insurance claims and encounter data from approximately 100 large employers and health plans in the US. Visit data include information on diagnoses, procedures, payment and whether the claim was paid as out-of-network. To ensure we have all information on mental health service use, we omitted plans that carved out mental health benefits. We limited our analyses to individuals continuously enrolled over the 6-year time period studied. Individuals ages 18–59 at baseline (2007) who used any mental health services during the study period were included (*N* = 698,680). We choose to study a continuously enrolled sample (i.e., the same underlying population in all years) to ensure that findings were due to changes in treatment rather than changes in the composition of the sample. This results in the cohort aging over the course of the study. We do not expect relatively small increases in age to have significant effects on out-of-network health care use among adults, the population studied here. We further limited the sample to individuals in self-insured plans. Self-insured plans had been exempt from prior state parity laws, making them all newly subject to parity under the federal law.

### Identifying mental health services

We considered all claims with a primary mental health diagnosis or a mental health-specific procedure code and inpatient stays with a mental health DRG to be mental health treatment. Diagnostic codes 295 through 309 (except 303, 304 and 305), and 311 through 314 in the *International Classification of Diseases, Ninth Revision, Clinical Modification (ICD-9-CM)* were considered mental health. Current Procedural Terminology (CPT) procedure codes specific to mental health treatment relevant during our study period include, for example, 90801 for a psychiatric interview examination, 90802 for individual psychotherapy, and 90862 for medication management. We considered DRG 876 through 897 to be mental health. Inpatient stays were considered out-of-network if the stay included either an out-of-network facility fee or professional services paid as out-of-network. We report all relevant outcomes in real 2012 dollars. Because prior work has found different responses to parity for substance use disorders and mental health, we considered the effects on substance use disorders in a separate paper [10].

### Outcomes

To study changes in out-of-network mental health service use, for each month of our study we calculated the proportion of patients who used an out-of-network provider among patients who used any mental health treatment that month. For the remaining mental health outcomes we limited the sample to individuals who used at least one out-of-network mental health provider during the relevant month. Because only two percent of patient-months included both in-network and out-of-network provider use, we categorized these patients as using out-of-network care rather than create a separate ‘mixed’ group. For each month, we calculated the mean number of outpatient out-of-network mental health visits and the mean total out-of-network mental health spending. The spending outcome includes payments by patients and insurers for both outpatient and inpatient use. For inpatient use, we considered all costs out-of-network if either the facility is out-of-network or the majority of professional service claims related to that stay are out-of-network. We did not include costs related to balance billing because these costs are not available in claims data.

### Statistical approach

Because federal parity was implemented nationwide, we did not have a valid comparison group that was unexposed to federal parity. Thus we used interrupted time series regression to predict expected outcomes in the absence of federal parity. In each model we included a continuous variable indicating the time in months since parity implementation, which ranges from -36 to +36 (*Time)*, a binary variable indicating whether the month is post implementation of federal parity, 2010 or later (*Parity*), an interaction of these two variables (Time * Parity) and 12 binary variables indicating calendar months. The coefficient on *Time* indicates the trend in the relevant outcome in the absence of parity, the coefficient on *Parity* indicates the level change in the outcome associated with parity implementation and the coefficient on Time * parity indicates the change in the baseline trend associated with parity implementation. The month indicators control for seasonality. For each outcome, one observation for each month of our study period is included (*N* = 72). We used Yule Walker first order autoregressive parameters to control for correlation between consecutive months. In alternate models we included a quadratic time trend. Results were similar across models. To ease interpretation of results, we used the estimated coefficients from this model to predict use and spending over the time period studied, and then estimated what the model predicted would have occurred in the absence of federal parity implementation and graphed these two values over time.

We conducted several sensitivity analyses. First, to determine whether any results are due to changes in medical benefit design rather than specific to mental health we use similar methods to consider changes in diabetes treatment. We expect no change in out-of-network diabetes treatment in response to federal parity. Second, we tested whether there were any additional changes in out-of-network service use and spending after the interim final parity regulations were implemented (in 2011) by adding a second binary parity variable indicating February 2011 and later, and interacting this variable with a second time variable.

## Results

Unadjusted demographic and clinical characteristics of the full sample of individuals using mental health services are included in Table 1. We separated individuals using only in-network mental health services from those obtaining either some or all of their mental health services out-of-network, and examined whether there were differences between these two groups. The group using only in-network services differs from the group using out-of-network services by region, with more representation of individuals from the South and West in the in-network group (*p* < .001). Individuals with psychosis, bipolar disorder, depression/anxiety and adjustment disorder have greater representation in the group using at least one out-of-network provider (*p* < .001).

**Table 1:** Unadjusted descriptive characteristics of continuously enrolled (2007–2012) individuals using mental health services in network and out-of-network before and after implementation of federal parity

	Full sample	Enrollees using only in-network services				Enrollees using some or only out-of-network services				
	2007–2012	All years 2007–2012	Pre-parity 2007–2009	Post-parity 2010–2012	*p*-value (pre vs post)	All years 2007–2012	Pre-parity 2007–2009	Post-parity 2010–2012	*P*-value (pre vs Post)	In-network vs OON 2007–2012
*N*	698,680	584,719	379,526	437,203		113,961	68,481	66,195		
Female (%)	62	62	63	63	0.019	62	63	62	0.004	0.289
Age (%)										
18–34	18	18	17	18	<0.001	18	18	18	0.128	<0.001
35–44	31	31	31	31	0.771	32	32	31	0.020	
45–54	37	37	37	36	<0.001	36	36	37	0.341	
55–64	14	14	14	14	<0.001	14	14	14	0.916	
Region (%)										
Northeast	11	11	11	11	0.020	14	15	15	0.195	<0.001
Midwest	26	24	24	25	<0.001	36	40	32	<0.001	
South	37	38	38	37	<0.001	33	30	34	<0.001	
West	26	27	27	27	<0.001	17	15	20	<0.001	
Lives in a designated MSA (%)	86	86	86	86	0.360	86	86	87	0.556	0.030
Diagnosis (%):										
Psychosis	1.6	1.1	0.9	1.1	<0.001	3.9	2.9	3.9	<0.001	<0.001
Bipolar Disorder	3.7	2.9	3.4	3.1	<0.001	8.3	7.8	8.1	0.021	<0.001
Depression or Anxiety	56.0	55.2	51.4	49.7	<0.001	59.9	56.5	56.2	0.222	<0.001
Attention-deficit hyperactivity disorder	2.9	2.9	2.9	3.4	<0.001	2.7	2.7	2.9	0.060	<0.001
Other	6.2	6.6	5.9	5.2	<0.001	3.7	3.6	3.8	0.098	<0.001
Adjustment disorder	14.2	14.2	13.4	12.9	<0.001	14.6	17.8	13.8	<0.001	<0.001
Residual	15.4	17.1	22.1	24.6	<0.001	6.9	8.7	11.2	<0.001	<0.001

Notes: Age, region and percent living in designated MSA are measured at baseline (2007). We used diagnosis codes to group patients into mutually exclusive hierarchical diagnosis categories. We required patients have the same ICD-9 code listed in the first diagnosis field on at least 2 claims on two dates to have a diagnosis. The diagnostic hierarchy was as follows: schizophrenia, bipolar disorder, depression or anxiety disorders, attention-deficit/hyperactivity disorder, other (including, for example, other affective disorders, other neurotic disorder, eating disorders), adjustment disorder, and a residual category including patients not included in the above

**Fig. 1:**
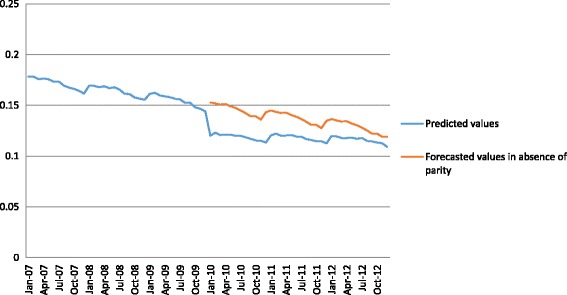
Share of mental health service users with any out-of-network mental health service use, 2007-2012. Notes: Predicted values (*blue line*) are derived using estimated coefficients to predict use and spending over the time period studied. Forecasted values in the absence of parity (*red line*) indicate model predictions of what would have occurred in the absence of federal parity implementation

Regression results are noted in Table 2. We found a significant one-time 3.1 percentage point decline in out-of-network service use at parity implementation – January 2010 (*p* < .001). Over our study period, there was a general decline in use of out-of-network providers, from 18 to 12% (Fig. 1; *p* < .001). Parity implementation was associated with a significant slowing of this trend such that after the initial drop in out-of-network service use at parity implementation, in the 3 years post parity, the rates of out-of-network provider use did not change.

**Table 2:** Interrupted Time Series Regression Results on the Probability of Out-of-network Mental Health Service Use, Quantity of Out-of-network Services, and Total Out-of-network Spending 2007–2012

	Coefficient	Standard Error	*P*-Value
Share with any out-of-network mental health service use among mental health service users			
Parity	−0.0305	0.001517	<0.001
Time	−0.0007	0.0000527	<0.001
Parity*Time	0.0006	0.0000744	<0.001
Average number of out-of-network mental health outpatient visits among out-of-network mental health service users			
Parity	0.1164	0.0211	<0.001
Time	0.0017	0.0071	0.019
Parity*Time	0.0053	0.0010	<0.001
Average total out-of-network mental health spending among out-of-network mental health service users			
Parity	49.1050	13.7305	0.001
Time	2.8294	0.4263	<0.0001
Parity*Time	−1.3412	0.6371	0.040

Notes

The unit of observation is the month/year (*N* = 72). All regressions include 11 dummy variables to control for month/seasonality

*P*-values reflect 2-sided tests

**Fig. 2:**
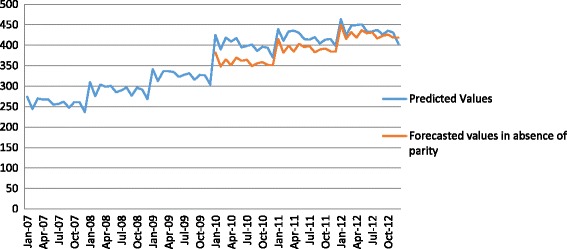
Average total OON mental health spending among out-of-network mental health service users, 2007-2012. Notes: Predicted values (*blue line*) are derived using estimated coefficients to predict use and spending over the time period studied. Forecasted values in the absence of parity (*red line*) indicate model predictions of what would have occurred in the absence of federal parity implementation

We found a significant increase in the average number of outpatient out-of-network mental health visits among out-of-network mental health service users at parity implementation. This increase was small in magnitude: 0.12 of a visit or approximately a 5% increase compared to 2007. We also found parity was associated with a small increase in the trend in the average number of out-of-network visits, approximately four-tenths of a visit per year. As indicated in Table 2 and Fig. 2, we found a significant $49 increase in average monthly out-of-network mental health spending among out-of-network mental health service users at parity implementation (*p* < .001). In the period before parity, there was a significant increasing trend in monthly out-of-network mental health spending of $2.83 per month (*p* < .001). Parity implementation was associated with a significant slowing of this trend (-$1.34 per month) (*p* < .05) such that spending was at the original pre-parity projected levels by the end of our study period.

As anticipated, we found that federal parity was not associated with changes in the share with out-of-network diabetes services use among users of diabetes-related care (see Additional file 1). In addition, we found no additional changes in out-of-network mental health service use or spending concurrent with implementation of the interim final regulations in February 2011 (see Additional file 1).

## Discussion

We examined the association of federal parity implementation with changes in use and spending on out-of-network mental health services. We found a significant 3.1 percentage point decline in the proportion of mental health patients who used at least one out-of-network service (from a baseline rate of 18%), which may suggest greater access to more affordable in-network providers. Among patients who used out-of-network services post-parity, we found a significant but small increase in the mean number of out-of-network mental health visits. We found an increase in total spending on out-of-network mental health services post parity. This one-time increase in spending was accompanied by restrained growth in spending, such that by the end of our study period there were no significant differences in monthly total spending on services out-of-network compared with what one would expect given existing pre-parity trends.

Our results indicating a decline in out-of-network service use post-parity were unexpected. Because we have limited ability to uniquely identify providers over time and do not have access to specific plan information related to network size, composition or reimbursement, we cannot identify with certainty the underlying mechanism driving these results. Other work suggests that the share of plans with higher out-of-network outpatient mental health copayments or coinsurance (compared to out-of-network medical/surgical) declined at parity implementation, with one study indicating a decline from 25 to 8% [11]. Generally, one would expect reductions in cost sharing to lead to increases in use. Yet, insurers may have responded to the requirement for equivalent out-of-network cost sharing by using other methods to encourage use of in-network providers (e.g., expanding provider networks, providing greater access to price transparency tools, or increasing linkages of patients to in-network providers). There is some evidence this occurred. One survey of plans’ early response to parity found that 80% of plans reported expanding their mental health provider network in the first year of parity implementation [12].

A priori, another reason one might have anticipated an increase in out-of-network service use is research suggesting recent sharp declines in whether psychiatrists were accepting new patients with private non-capitated insurance [13]. Yet, in one study of the effects of a state parity law in Oregon, only 4–9% of mental health patients initiated treatment with a psychiatrist [14], indicating changes in psychiatrists’ participation in networks may have limited effects on out-of-network utilization overall, since such a high proportion of patients see other provider types. As suggested above, plans may have increased the number of non-psychiatrist mental health providers in networks in response to parity implementation. Finally, the fact that we do not find any additional significant changes in use and spending at implementation of the interim final regulations (in 2011) suggests the effects found are not due to the requirement that plans use the same standards for network participation or provider reimbursement for mental health and medical/surgical care. Future research should consider whether changes found in out-of-network care use are due to changes in provider network size or composition, reimbursement rates or other plan characteristics.

This study has several important limitations. First, our study does not use a comparison group and a concern with the interrupted time series methodology is that secular changes in mental health treatment or out-of-network service use unrelated to federal parity could drive results. It is challenging to identify an appropriate comparison group in the context of a national policy change implemented at a single point in time. We mitigate this concern by controlling for pre-parity trends in use and spending. That we do not find significant changes in studied outcomes at other time periods during our study period further increases our confidence these effects are related to federal parity. In addition, if our findings were being driven by secular trends in out-of-network service use, we would have expected to see similar trends for diabetes care. A second limitation is that changes found in number of visits and spending may reflect a compositional change in the group using out-of-network services, rather than a change for individual patients. If patients requiring more complex treatment were more likely to continue to use out-of-network services post parity implementation, average number of visits, and total spending would both likely increase. However, we do not find large changes in the distribution of diagnoses post-parity implementation, suggesting this is not driving our results. Third, we do not distinguish between new and on-going episodes of treatment when examining out-of-network care use. It may be that some of the care observed out-of-network post-parity was begun (and a provider chosen) in earlier periods. Fourth, we choose to study a continuously enrolled sample to avoid biases due to changes in the underlying population, this may possibly lead to other biases if age was associated with out-of-network use. Yet, we did not find meaningful differences in the age distribution comparing in-network versus out-of-network users (Table 1). Finally, the use of claims data to identify diagnoses and treatment can be problematic. *Truven Health MarketScan* is the only data we are aware of available to researchers that contains data from multiple insurers and notes whether a service was paid out-of-network for a large number of patients.

## Conclusion

There was concern that requiring equal coverage for out-of-network services under federal parity would reduce insurers’ ability to manage utilization and lead to large increases in out-of-network service use. This study suggests that out-of-network mental health service use and spending did not substantially increase post parity implementation, and these insurer concerns were unfounded.

## Additional file

Additional file 1: Table S1. Interrupted Time Series Regression Results on the Probability of Out-of-network Diabetes Service Use, 2007–2012. **Table S2** Regression results testing for additional effects of interim final regulation implementation in 2011 (DOCX 13 kb)

## Abbreviations

MHPAEA: Paul Wellstone Pete Domenici Mental Health Parity and Addiction Equity Act of 2008
CPT: Current procedural terminology
