# Supplementary material for: Was federal parity associated with changes in Out-of-network mental health care use and spending?
Source: BMC Health Serv Res. 2017 May 2;17:315. doi: 10.1186/s12913-017-2261-9 (PMC5414372; doi:10.1186/s12913-017-2261-9)
Supplement: Additional file 1: Table S1. — Interrupted Time Series Regression Results on the Probability of Out-of-network Diabetes Service Use, 2007–2012. Table S2 Regression results testing for additional effects of interim final regulation implementation in 2011 (DOCX 13 kb) [file 12913_2017_2261_MOESM1_ESM.docx]

**Additional file 1**

Interrupted Time Series Regression Results on the Probability of Out-of-network Diabetes Service Use, 2007-2012.

|  | **Coefficient** | **Standard Error** | **P-Value** |
| --- | --- | --- | --- |
| **Any out-of-network service use,**  **Diabetes sample (outpatient only)** |  |  |  |
| Parity | -0.0014 | 0.002138 | 0.518 |
| Time | -0.0001 | 0.0000733 | 0.165 |
| Parity*Time | -0.0002 | 0.000103 | 0.115 |

Notes:

All regressions include eleven dummy variables to control for month/seasonality.

P-values reflect 2-sided tests.

Regression results testing for additional effects of interim final regulation implementation in 2011

|  | **Coefficient** | **Standard Error** | **P-Value** |
| --- | --- | --- | --- |
| **Any out-of-network service use** |  |  |  |
| Parity | -0.0307 | 0.0021 | <0.001 |
| Month | -0.0073 | 0.0001 | <0.001 |
| Parity*Month | 0.0006 | 0.0003 | 0.0315 |
| Parity2 (2011) | 0.0020 | 0.0029 | 0.4832 |
| Parity2*Month | -0.00002 | 0.0003 | 0.9136 |
|  |  |  |  |
| **Number of out-of-network outpatient visits** |  |  |  |
| Parity | 0.0701 | 0.0335 | 0.0410 |
| Month | 0.0018 | 0.0070 | 0.0111 |
| Parity*Month | 0.0117 | 0.0040 | 0.0047 |
| Parity2 (2011) | 0.0598 | 0.0408 | 0.1492 |
| Parity2*Month | -0.0072 | 0.0040 | 0.0786 |
|  |  |  |  |
| **Total out-of-network mental health spending** |  |  |  |
| Parity | 57.2033 | 21.1207 | 0.0090 |
| Month | 2.7762 | 0.4427 | <0.0001 |
| Parity*Month | -3.4543 | 2.7662 | 0.2171 |
| Parity2 (2011) | 16.1035 | 25.3956 | 0.5287 |
| Parity2*Month | 1.3168 | 2.7993 | 0.6400 |
